# What European Union in the “Age of Uncertainty”? Weathering the Geopolitical Storms in a World of Perpetual Crises

**DOI:** 10.1007/s10272-022-1088-8

**Published:** 2022-12-16

**Authors:** Isabelle Ioannides

**Affiliations:** Hellenic Foundation for European & Foreign Policy, 49, Vasilissis Sofias Ave., 106 76 Athen, Greece

**Keywords:** P51, F61, Q20

## Abstract

A world of risks and uncertainties can also be a world of opportunities. How and how quickly the EU adapts to the socio-political, economic, energy and climate transitions at hand, and how it responds to geopolitical shifts and shocks, will determine whether it can sustain its role and standing on the international stage.

We live in times of uncertainty and unpredictability. Increasing tensions and competition between the United States and China are some of the many features mapping the global geopolitical landscape. Yet, despite a new Cold War seemingly settling in, the course of world politics will not be determined simply by the interaction between great powers. Rather, a more complex international order has emerged, where dispersed and contested multipolarity – as the UN General Assembly resolutions on the war in Ukraine have made obvious – will likely spur greater fragmentation. The war in Ukraine has demonstrated how countries, particularly key middle powers, can waver (uneasily) between sometimes acting constructively but other times adopting a neutral stance (e.g. Turkey) or initially aligning against the majority but then edging closer to a more neutral position (e.g. India). Regional heavyweights, such as Turkey and Saudi Arabia, have already been playing a pivotal role in shaping their regional contexts. Equally, South-South trade is bringing new patterns of interdependence and shifting the balance of power.

In this "age of uncertainty" (Galbraith, 1977), the way the diverse poles behave in a given crisis will define the trenches of each battle. Similar to Flockhart's (2016) "multi-order world", in an international system of several different "orders" (or international societies), the challenge will be to forge new forms of relationships between composite and diverse actors, which will not fall nicely behind clear fault lines. While the liberal order may continue, and could even be strengthened internally, its global reach belongs to the past. More critically, temporary and strategic alliances, driven by interests more than values, will be forged across complex lines of division and convergence. Thus, the world order will not be clearly cut into two blocs: one under US influence and another under Chinese influence.

Even before Russia's aggression against Ukraine and the coronavirus crisis, signals of the advent of a more competitive and less secure international geopolitical environment were visible. A 2013 study commissioned by the European Strategy and Policy Analysis System had warned about the dichotomy between a world of competition and cooperation becoming an especially acute challenge, power shifts becoming less linear, and power shifting to the East (in science and innovation, but also likely in military capacity) (Grevi et al., 2013).

## The revival of geopolitics

Geopolitics has caught up with Europe and found it rather unprepared, accelerating many pre-existing trends. The rise of authoritarianism has become more widespread globally. Only 6.4% of the world population (21 countries) lives in full democracies, according to the latest Democracy Index. The rest live in flawed democracies (almost 40%, 53 countries), hybrid regimes (17%, 34 countries) or authoritarian regimes. In fact, more than a third of the world population (37%, 59 countries) lives in authoritarian countries (Economist Intelligence Unit Ltd., 2022a).

China and Russia's authoritarianism has stretched to certain EU aspiring members in the Western Balkans, while some EU member states have veered towards illiberalism. Beyond the populist trends in Hungary and Poland, the recent victory of Giorgia Meloni in Italy and the rise of the far right in Sweden illustrate the societal discomfort in our Western democracies and the failure of politics to meet citizens' expectations and own up to promises made. Nevertheless, despite the rise of the radical right and populists at the ballot box in Europe, these movements have managed to gain effective power only where they have controlled the executive branch of government (e.g. in Hungary). Where populist parties have found themselves in coalitions, across a vibrant political opposition and an active civil society, as is the case of Poland, their power has been kept in check. However, the rise of populism will likely remain a challenge in Europe and will require vigilance.

The West's systemic rivalry with Russia and China has intensified in recent years. Beyond the normative confrontation that underpins Russia's war in Ukraine, a territorial war has unexpectedly been brought back on the European continent. China too has become an even stronger global competitor for the EU, the United States and other like-minded partners. While China appears to have somewhat distanced itself from Putin's objectives in Ukraine recently, especially from Russian threats to use nuclear weapons, China-Russia bilateral relations increasingly look like a strong strategic partnership that cannot be ignored. In this context, some have called for the EU's formula "partner-competitor-rival", as it appeared in the 2019 EU strategy on China, to be replaced by "competitor-rival-partner" (Lau, 2022).

Russia's aggression against Ukraine and the coronavirus crisis have further accelerated and diversified the hybrid wars underway. Firstly, the backlash of technology has further intensified, manifested by disinformation against state and non-state entities affecting electoral processes, as well as through online smear campaigns against a wide range of actors, from human rights defenders and democracy workers to political institutions, inter-governmental organisations and politicians. The recent ethnic tensions in North Kosovo, fuelled by nationalist rhetoric from Belgrade and by Russian disinformation, are a case in point.

Secondly, the weaponisation of energy has revealed Europe's vulnerabilities. On the one hand, the EU has been dependent on Chinese and Russian resources. Russia was the leading supplier of the main fossil fuels to the EU (oil, gas, coal) over the 2010-2020 period (Eurostat, 2022). In 2021, the EU was Russia's top energy client having imported 27% of its oil and 47% of its coal from there. Although oil and gas imports from Russia have dropped remarkably since Russia's invasion of Ukraine, they are only slowly being replaced by substitutes from the United States and Norway (Vistesen and Debono, 2022). Equally, shortages of gold, silver and minerals have affected such EU industrial sectors as batteries, electronics, automotive and machinery (EPRS, 2022). Europe alone is more than 90% dependent on China for mined or processed minerals needed for the energy transition. On the other hand, the energy transition could also render the EU dependent on new major players on the energy scene that are outside of the EU's sphere of influence. Morocco, Egypt, Namibia and the Gulf region will likely play a key role in the hydrogen, sun and wind sectors. Countries that possess critical minerals, e.g. Chile (copper) and China (rare earth), will also be crucial potential EU partners (Leruth and Mazarei, 2022; Borrell, 2021; Mills, 2022). Equally, Russia is quickly expanding its drills of oil, gas and minerals across the African continent, while China dominates the global supply of critical raw materials (Economist Intelligence Unit Ltd., 2022b).

Thirdly, Russia's invasion of Ukraine has brought to the fore the geopolitics of food. Russia and Ukraine provided over one-third of the global wheat, maize and sunflower exports before the war. Approximately 90% of Ukraine's wheat exports went to Africa and Asia between 2016 and 2021, supporting food security in some of the most disadvantaged regions of the world. The ongoing war in Ukraine threatens fragile, poor and vulnerable countries with important food shortages, a situation further exacerbated by the rise of global inflation. According to the FAO Food Price Index, Eastern African and Middle Eastern and North African countries, which import respectively 90% and over 50% of their cereals from Ukraine and Russia, are among the most impacted (Boehm et al., 2022). With Russia's blockade of Ukraine's ports from the beginning of the war, around 20 million tonnes of grain were stuck in storage silos on the Black Sea shores. While 61% of Ukraine's food exports (2.6 million tonnes) have since been transported via the solidarity lanes that the EU established in May (European Commission, 2022),[1] in reality, the EU's role at the political level to broker the grain deal has been limited.

The climate crisis has also caught up with the EU. Climate change has been a challenge distressing Africa for at least the past 20 years. But now that compounding natural disasters in Europe have affected citizens and the EU economy directly, climate policy has increasingly found itself on the EU agenda. The World Meteorological Organization has found a disconcerting trend of increased warming across Europe at a rate that is not only faster than on any other continent, but also twice as fast as the global average. Average temperatures in Europe were rising at a rate of 0.5 degrees Celsius (0.9 degrees Fahrenheit) per decade between 1991 and 2021, reaching an overall average of 2.2 degrees C (4 degrees F) above pre-industrial levels. That is well above the 1.5 degrees C (2.7 degrees F) limit on the global average temperature rise set by the Framework Convention on Climate Change (Gohd, 2022; Pultarova, 2022).

## A transformative moment for the EU?

The new realities of the international order and the geopolitical shifts and shocks have made the urgency for the EU to adapt evident. The Russian invasion of Ukraine has revitalised NATO and united Europeans. But this will not be enough. How the EU will manage its relationship with China will be key, in full knowledge that the EU's normative agenda sits squarely with the United States and NATO, the EU's reliable and fundamental partners.

The EU was built for a world that is peaceful, multilateral and driven by compromise. Already in its first European Security Strategy in December 2003, the EU had set "effective multilateralism" – defined as "the development of a stronger international society, well-functioning international institutions and a rule-based international order" – as one of its three strategic objectives (Council of the EU, 2003). Strengthening the European Union's role as a credible global actor by "champion[ing] multilateralism and a rules-based global order through a more active role and stronger voice for the EU in the world" is also one of the ten priorities of President von der Leyen's "geopolitical Commission" (European Commission, 2022b). Trade has been one of the EU's strongest instruments and after a

1 The solidarity lanes aim to provide alternative overland routes to help Ukraine export its agricultural products.

period of standstill during the coronavirus pandemic, the EU is re-launching trade negotiations with a number of key partners (e.g. India, Japan, Australia and New Zealand). The EU will want to use other strong aspects of its rule-based portfolio (e.g. the single market and its regulatory framework that sets standards) to propel itself on the global scene.

The world that the EU was built for no longer exists. The avalanche of crises that have hit Europe and the world and weakened multilateralism have created circumstances so tangible that political leaders cannot hide from them, nor can they hide from their electorate. That also became apparent in the debates organised at the Conference on the Future of Europe (2022). Nevertheless, the EU has proven in the past that crisis can be an instigator for structural and functional change, even if such change is uneven and incomplete (see for example Anghel and Jones, 2022; Jones et al., 2016). The EU has also become a faster crisis manager: while it took the EU years to tackle the financial crisis, it reacted in months to the coronavirus pandemic, and only days to Russia's aggression against Ukraine. Nevertheless, while it is unlikely that "Europe's coming of age" is coming (Tsoukalis, 2022), this needs to be a transformative moment for the EU – one in which the EU goes beyond its instrument-driven approach to become a more autonomous and strategic actor.

## Boosting European sovereignty

French President Emmanuel Macron's 2017 call for "European sovereignty", taken up by the EU in 2020 as "strategic autonomy" (a catchphrase for the EU's push to increase self-sufficiency and boost its own capabilities) is more of a political imperative today than ever before. The war raging on the EU's eastern border has exposed the limits of the EU's soft power model, and therefore, those of the most narrow interpretation of European sovereignty, that of EU defence and security. In reaction, the EU has imposed unprecedented sanctions on Russia and has supported Ukraine with substantial arms deliveries. For the first time ever in EU crisis management, its military aid outweighs its humanitarian aid package. This is the result of clear EU political will, i.e. member states managing to muscle consensus quickly at a critical moment.

Moving more decisively towards the development of military capacities in the short term and a real European Defence Union in the long term will become a recipe for survival. China's industrial capacity and defence spending are expected to overtake those of the United States by 2030. Moreover, resource-dependent rising powers, including India (in addition to China), are set to continue to increase their commercial, political, even military presence beyond their immediate regions (Grevi et al., 2013). In fact, the manufacturing competitiveness of China, India and South Korea has not only led to the growth of regional production networks, but has also reconfigured the global industrial landscape (including in new technologies). In parallel, Russia is expanding to the Sahel region of Africa by using the Wagner group to capture the vacuum left behind in Mali by France, but also in the Central African Republic and Libya.

EU defence cooperation has, especially since 2016, progressively deepened, and funding is increasing. However, this process has been slow and shy. The recent Coordinated Annual Review on Defence revealed that defence cooperation remains the exception rather than the rule. According to the European Defence Agency, "defence planning continues to take place mainly in isolation and Member States are still not convinced by European cooperation projects" (European Defence Agency, 2022a). In this vein, 52% of defence investments are national and only 18% are collaborative, while the target set in the Strategic Compass is at 35% (Agence Europe, 2022). The main drivers of cooperation appear to be national end-benefits, cost savings, strengthening strategic partnerships, and increasing operational efficiency. Cooperation is primarily limited to joint ventures between neighbouring states within existing cooperation frameworks, while broader European collaborative approaches are generally not preferred (European Defence Agency, 2022b). It will be up to EU member states to radically revise how they think and act on their multi-billion-euro national defence programmes (Ioannides, 2020a).

Equally, intelligence at the EU level has been the weakest link in its crisis management action. EU engagement in the Sahel region is a case in point. When the EU was reflecting on how to organise its action on the ground, it had to do so in full cognisance of statements by then US President Donald Trump about the US withdrawal from the Sahel. The closure of the major US drone base in Niger would leave a critical intelligence vacuum (Ioannides, 2020b). This mismatch in intelligence continues and was exposed again when Russia invaded Ukraine, which caught the EU off guard despite the warning from the United States. Regardless, the EU is still heavily dependent on US intelligence for organising its war support for Ukraine.

The EU will also need to leapfrog into the twin transition – digital and green – and invest in advanced technologies to shore up infrastructure resilience and guard against disruption. As EU sanctions become increasingly severe and the West continues to exclude Russia from the global economy, experts expect that Russia will struggle to finance current and prospective mining operations in Africa, a vacuum in the market that the EU could potentially usurp. Russia's growing mining activities in Africa have concentrated in weakly governed and authoritarian states (e.g., Sudan, Guinea, the Central African Republic and Mozambique). However, some of Russia's partners are in the EU's neighbourhood and are thus obvious EU counterparts (e.g., Morocco and Algeria) (Economist Intelligence Unit, 2022b).

Figure 1

**Defence expenditure as a share of GDP**

in %, based on 2015 prices and exchange rates

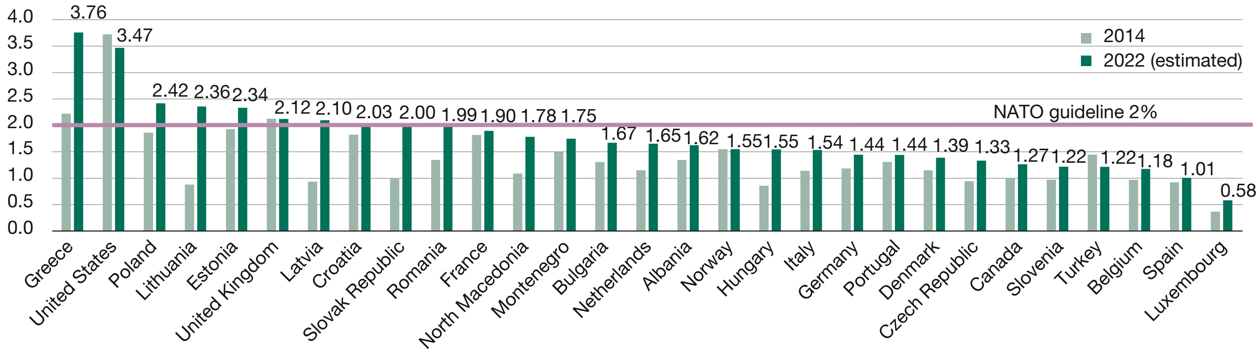

Source: NATO, Defence Expenditure of NATO Countries (2014-2022), Communique PR/CP(2022)10527, June 2022, 3.

The war in Ukraine and the realisation of the urgency to push forward with European sovereignty has put EU enlargement back on the EU agenda, a process that has been on the backburner for the last 15 years or so. EU enlargement is seen as a way to counter Europe's demographic downsizing, but could also help the EU expand its orbit of influence to the entire continent (Dimitrov et al., 2022). Moving towards a wider Europe of 35 (including the six Western Balkan countries, Ukraine and Moldova) or 36 (also with Georgia) and even 37 (if the United Kingdom were to reintegrate into the EU) can no longer be discarded.

## Reducing the EU-US capability gap

Boosting European sovereignty would also help the EU create a less asymmetrical relationship with the United States. While the EU cannot afford to vacillate between the United States and China, it will need to reduce the capability gap with the United States to bridge the geopolitical rifts that threaten its long-term competitiveness. European sovereignty should not only boost the EU's prosperity that is at risk, but also bring more legitimacy to the transatlantic relationship. US financial and military support for Ukraine vastly exceeds that of the EU, and as the United States pivots to Asia to meet the challenge from China, it laments that the EU fails to pay for its own security. Most EU members that are also NATO members have yet to meet the goal of spending 2% of their GDP on defence, as shown in Figure 1.

Geopolitical realities have meant that boosting economic security has become a paramount priority for both the EU and the US. However, a divide over how to best accomplish this is increasingly visible across the Atlantic. While European leaders still grapple with how to stay competitive while strengthening resilience and autonomy, US leaders increasingly appear concerned above all with curbing China's technological rise and economic reach by breaking up supply chain concentrations. In this light, the CHIPS and Science Act marks a spectacular revival of US industrial policy at the service of a geoeconomic goal: winning the technology competition with China (Gehrke, 2022). It does so by providing some $52 billion in subsidies for the semiconductor industry (and some $170 billion into R&D and STEM education), thus exemplifying Washington's hardening view on investments in Chinese manufacturing.

In this same logic of seeking to boost the US industrial base, the Inflation Reduction Act aims to curb inflation by investing in domestic energy production while promoting clean energy. It does so partly by offering subsidies only to US-manufactured electrical vehicles. This has upset a number of the United States' trading partners – not only the EU but also Japan, South Korea and Canada – who perceive this measure as unfair competition. The EU has managed to get its relationship with the US back on the rails since the election of President Joe Biden, an EU-US task force has been set up to deal with such potential disputes, and the West will want to show a united front on the global scene. Yet, the subsidy row carries elements of a trade dispute and feeds tensions between the US and Europe. Resolving the dispute could be complicated given how sensitive the subsidy issue is domestically in the United States, as it was both a huge political win for Biden and a difficult balancing act to get it through Congress.

## Capitalising on the EU consensus experience

The EU could build and capitalise on its political culture of consensus. By consolidating European values and protecting its model internally, a model that has become increasingly difficult to export to third countries, the EU could play a crucial stabilising role in this unstable period and emerging world order. However, as the energy crisis rages, the war in Ukraine has exposed a vulnerability in Europe's business model, especially in Germany, where industrial firms have relied on abundant energy inputs from Russia and are dependent on China as an end market.

The strong Franco-German partnership has been an engine for negotiating unity of purpose to uphold EU interests and values, especially following Brexit. This role could be key to guiding internal EU reforms and steering EU external action. Yet today's global competition has become a factor for divergences of interests between the two EU member states on energy subsidies, gas price caps and joint defence projects. France, a nuclear power with a seat at the UN Security Council and more alternative energy sources than Germany, is less worried about its strategic autonomy and more about its economic sovereignty (Attali, 2022). Germany is looking to retain its economic sovereignty, which is compromised by its decoupling from Russia on energy and could be further hit if it cuts supply chains with China. At the same time, German Chancellor Olaf Scholz is looking to build closer connections with other Asian partners (e.g. Japan and India), in line with his worldview of multiple emerging global powers, and the need to foster resilience, diversification and "de-risking" in achieving economic security vis-à-vis China (Benner, 2022). To boost its defence capabilities, Germany tends to look over the Atlantic, to the United States.

Nevertheless, given the EU's emphasis on rules-based cooperation, its consensus-building experience in decision-making could be an advantage in a more competitive international system with more assertive powers. It therefore could play a role in negotiating relations with middle powers, either through traditional means (e.g. partnering with key states or deepening security dialogues) or through more innovative avenues (e.g. developing plurilateral security negotiating forums). This could be a way of developing relations on a functional basis with selected partners on such diverse issues as counterpiracy, cybersecurity, maritime and resource security and state fragility. While Beijing and Moscow enjoy robust political ties, their relations are fraught with contradictory economic interests in Central Asia, particularly on the region's natural gas exports that are seen as potentially impeding Gazprom's ability to supply the Chinese market with natural gas. Despite Central Asian governments needing to walk a tightrope between their two giant neighbours, the war in Ukraine has eroded Putin's power in Central Asia and therefore improved prospects for the region's natural gas exports to other major players, including to the EU (Palanch and Glaser, 2021).

A world of risks and uncertainties can also be a world of opportunities. How and how quickly the EU adapts to the socio-political, economic, energy and climate transitions at hand, and how it responds to geopolitical shifts and shocks, will determine whether it can sustain its role and standing on the international stage.
